# Differential proteomes of the cyanobacterium *Cyanothece* sp. CCY 0110 upon exposure to heavy metals

**DOI:** 10.1016/j.dib.2015.04.015

**Published:** 2015-05-22

**Authors:** Rita Mota, Sara B. Pereira, Marianna Meazzini, Rui Fernandes, Arlete Santos, Caroline A. Evans, Roberto De Philippis, Phillip C. Wright, Paula Tamagnini

**Affiliations:** a Instituto de Investigação e Inovação em Saúde, Universidade do Porto, Portugal; b IBMC - Instituto de Biologia Molecular e Celular, Universidade do Porto, Porto, Portugal; c Faculdade de Ciências, Departamento de Biologia, Universidade do Porto, Porto, Portugal; d Department of Agrifood Production and Environmental Sciences, University of Florence, Florence, Italy; e ChELSI Institute, Department of Chemical and Biological Engineering, University of Sheffield, Sheffield, United Kingdom; f Institute of Ecosystem Study (ISE), National Research Council (CNR), Sesto Fiorentino (FI), Italy

**Keywords:** Cadmium, Copper, Cyanobacteria, *Cyanothece*, iTRAQ, Proteome

## Abstract

The proteomes of the highly efficient extracellular polymeric substances (EPS)-producer cyanobacterium *Cyanothece* sp. CCY 0110, grown in medium supplemented with an essential metal (Cu^2+^) or a non-essential metal (Cd^2+^),were compared using iTRAQ technology. The data were obtained within a larger study that evaluated the overall effects of different heavy metals on growth/survival, EPS production and ultrastructure of this cyanobacterium [1]. To allow a broader understanding of the strategies triggered to coupe with toxic effects of the metals, *Cyanothece*′s proteomes were evaluated after chronic and acute exposure to Cu^2+^ and Cd^2+^ in two independent 8-plex iTRAQ studies. For the chronic exposure 0.1 mg/l of Cu^2+^ or 5 mg/l of Cd^2+^ were used for 10 and 20 days, while in the acute experiments the cells were exposed to 10× these concentrations for 24 h. 202 and 268 proteins were identified and quantified for studies 1 (Cu^2+^) and 2 (Cd^2+^), respectively. The majority of the proteins with significant fold changes were associated with photosynthesis, CO_2_ fixation and carbohydrate metabolism, translation, and nitrogen and amino acid metabolism.

Specifications Table

**Table:** 

Subject area	Biology
More specific subject area	Cyanobacterial proteomics
Type of data	Figures, excel files
How data was acquired	iTRAQ labelling (AB SCIEX^™^);
	Ultimate 3000 HPLC (Thermo Scientific) with an PolyHydroxyethyl^™^ A column (PolyLC);
	QStar XL Hybrid ESI Quadrupole Time-of Flight Mass Spectrometer (ESI-qQ-TOF-MS/MS; AB SCIEX^™^) coupled to an Ultimate 3000 HPLC (Dionex) with an Acclaim^R^ PepMap100 C18 column (Thermo Scientific);
	Analyst QS 1.1.1 (AB SCIEX^™^) coupled to mascot.dll embedded script (V1.6); Phenyx v2.6 (GeneBio S.A.) and UniProt database comprising all *Cyanothece* sp. CCY 0110 protein sequences (6413 entries, March 2014).
Data format	Filtered, analysed
Experimental factors	Protein samples were denatured with SDS, reduced with TCEP, alkylated with MMTS and digested with trypsin and labelled with iTRAQ reagents according to manufacturer׳s procedures.
Experimental features	After iTRAQ labelling and combining of samples, high-resolution hydrophilic interaction chromatography (HILIC) fractionation was performed. Fractions were cleaned using C18 UltraMicroSpin Columns (The Nest Group Inc.) according to the manufacturer׳s guidelines before reverse phase liquid chromatography (RPLC)–MS/MS analysis. Raw data was searched on MASCOT for protein identification. Protein quantifications were carried out computing the geometric means of the reporters׳ intensities using an in house data analysis pipeline. Proteins were organised into functional groups according Gene Ontology information available in Uniprot.
Data source location	Porto, Portugal and Sheffield, United Kingdom
Data accessibility	Analysed data sets directly provided in this article

Value of the data

• Effects of heavy metals on the strong EPS-producer *Cyanothece* sp. CCY 0110.
• Differential proteomes in medium/medium supplemented with heavy metals.
• Specific effects related to time of exposure and/or concentration of the metal.
• Short- and long-term strategies to coupe with metal toxic effects.

## 1 Data

**Fig. 1:**
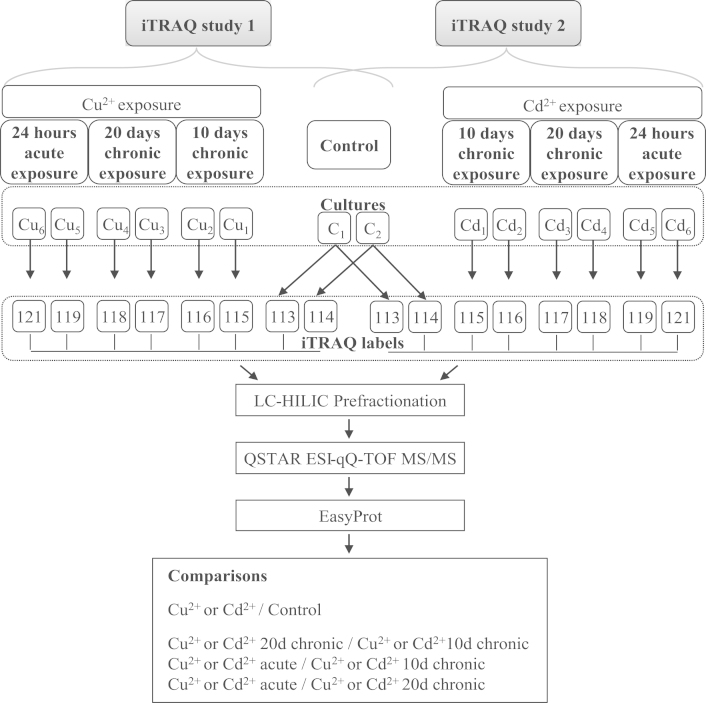
Graphical representation of the iTRAQ workflow and analyses preformed.

The proteomes of *Cyanothece* sp. CCY 0110 grown in medium or medium supplemented with Cu^2+^ or Cd^2+^ were compared using two independent 8-plex iTRAQ studies (Fig. 1). For the chronic exposure, sub-lethal concentrations of 0.1 mg/l of Cu^2+^ or 5 mg/l of Cd^2+^ were used for 10 and 20 days, while in the acute experiment the cells were exposed to 10× these concentrations for 24 h. In total, 202 (98 with two or more peptides) and 268 (130 with two or more peptides) proteins were identified and quantified for iTRAQ study 1 – Cu^2+^ and iTRAQ study 2 – Cd^2+^, respectively. The complete lists of peptides and proteins identified in iTRAQ studies 1 and 2 are provided in Supplementary Tables 1 and 2 respectively, and protein quantifications are provided in Supplementary Table 3.

**Fig. 2:**
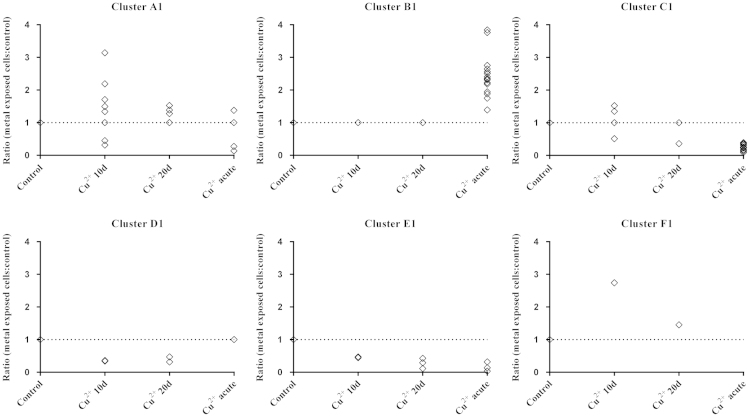
Hierarchical cluster analysis of the proteins quantified in iTRAQ study 1 (Cu^2+^ exposure). Six (A1-F1) clusters of proteins were defined according to the variation of their relative levels in *Cyanothece* cells grown in ASNIII buffered medium supplemented with 0.1 mg/l of Cu^2+^ (for 10 or 20 days, chronic exposure) or 1 mg/l of Cu^2+^ (24 h, acute exposure). Clusters were calculated using all ratios to minimise over- or underestimations. Data were converted into ordinal/ranked variables and clustered using the “centroid linkage” method and the “squared Euclidean distance” measure.

**Fig. 3:**
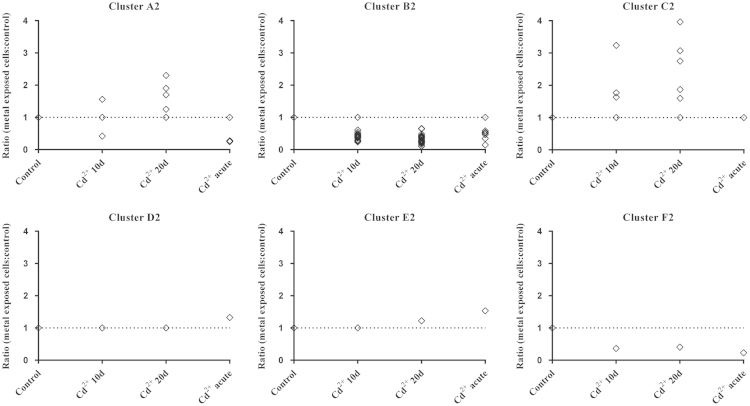
Hierarchical cluster analysis of the proteins quantified in iTRAQ study 2 (Cd^2+^ exposure). Six (A2–F2) clusters of proteins were defined according to the variation of their relative levels in *Cyanothece* cells grown in ASNIII buffered medium supplemented with 5 mg/l of Cd^2+^ (for 10 or 20 days, chronic exposure) or 50 mg/l of Cd^2+^ (24 h, acute exposure). Clusters were calculated using all ratios to minimise over- or underestimations. Data were converted into ordinal/ranked variables and clustered using the “centroid linkage” method and the “squared Euclidean distance” measure.

To identify groups of proteins (clusters) with similar variation patterns, hierarchical cluster analyses were performed. The strength of the analyses was improved by taking into account the ratios obtained for metal-exposed conditions compared to control, as well as those resulting from the comparison of different metal-exposed conditions (Fig. 1). This approach minimises the effects of over- or underestimated ratios and increases confidence. For each iTRAQ study, six statistically supported protein clusters (A–F) were formed (Figs. 2 and 3). Regarding study 1, 80% of proteins was included in cluster A1 (no significant change in any of the conditions tested), cluster B1 (no significant change in 10 and 20 days chronic exposure, and higher abundance in acute exposure) and cluster C1 (no significant change in 10 and 20 days chronic exposure, and lower abundance in acute exposure) (Fig. 2). Overall, the acute exposure of Cu^2+^ was the condition that promoted more quantitative proteome changes – 19%. Concerning study 2, 87% of the proteins were found in cluster A2 (no change in any of the conditions) and cluster B2 (lower abundance in 10 and 20 days chronic exposure) (Fig. 3). In contrast with what was observed for Cu^2+^, in study 2 the 10 and 20 days chronic exposure were the conditions that caused more differential protein expression, 12% and 13% respectively.

To gain insight into the biological significance of the changes observed, the proteins were grouped according to their annotated function and the Gene Ontology information [1]. The majority of the proteins with known functions were associated with photosynthesis, CO_2_ fixation and carbohydrate metabolism, translation, and nitrogen and amino acid metabolism.

Overall, the results obtained suggest that during Cu^2+^chronic exposure the cells adjust their metabolism to invest the spare energy in the activation of metal detoxification mechanisms. In contrast, the toxic effects of Cd^2+^accumulate over time suggesting that cells might not have the same capacity to deal with this non-essential metal.

## 2 Experimental design, materials and methods

### 2.1 Organism and culture conditions

The unicellular cyanobacterium *Cyanothece* sp. CCY 0110 (Culture Collection of Yerseke, The Netherlands) was grown in 100 ml Erlenmeyer flasks containing ASNIII medium [2] (control) or in medium supplemented with 0.1 or 1 mg/l of copper (Cu^2+^, stock solution 10,000 mg/l in 1% HNO_3_, Sigma-Aldrich Co., MO, USA), or 5 or 50 mg/l of cadmium (Cd^2+^, stock solution 10,000 mg/l in 5% HNO_3_, Sigma-Aldrich). All cultures were buffered with 1 M MOPS (pH 7.0), grown at 30 °C under a 12 h light (50 µE/m^2^/s)/12 h dark regimen and with magnetic stirring (150 rpm).

### 2.2 iTRAQ experimental design

The experiments comprised two biological replicates for each 8-plex iTRAQ independent experiment. Two iTRAQ studies were performed (Fig. 1), namely the comparison of the proteomes of *Cyanothece* grown in the absence or presence of copper (iTRAQ study 1) or cadmium (iTRAQ study 2). The biological replicates used as control were common to the two studies. Both studies comprised four phenotypes of cells grown:

(C_1_, C_2_) in ASNIII buffered medium for 10 days (control).
(Cu_1_, Cu_2_ and Cd_1_, Cd_2_) in medium supplemented with either 0.1 mg/l of Cu^2+^ or 5 mg/l of Cd^2+^ for 10 days (chronic exposure).
(Cu_3_, Cu_4_ and Cd_3_, Cd_4_) in medium supplemented with either 0.1 mg/l of Cu^2+^ or 5 mg/l of Cd^2+^ for 20 days (chronic exposure).
(Cu_5_, Cu_6_ and Cd_5_, Cd_6_) in medium supplemented with either 1 mg/l Cu^2+^ or 50 mg/l Cd^2+^ for 24 h (acute exposure).

### 2.3 Protein extraction and quantification

The cells were harvested by centrifugation (3850 *g* for 15 min at room temperature), washed with buffer (50 mM Tris, pH 7.4, 100 mM EDTA, pH 8.0, and 25% (w/v) sucrose) and re-suspended in phosphate buffer (50 mM K_2_HPO_4_, 50 mM KH_2_PO_4_, pH 6.8). The proteins were extracted using the FastPrep^R^-24 cell disruptor, output 6.5 m/s, 5 cycles of 30 s (MP Biomedicals, LCC, CA, USA) and glass beads (425–600 µm, Sigma-Aldrich) for mechanical cell disruption, followed by centrifugation at 16,000 g for 15 min at 4 °C. The supernatant containing the soluble proteins was recovered and stored at −80 °C. The protein concentration was measured using the BCA^™^ Protein Assay Kit (Pierce Biotechnology, Inc., IL, USA) and iMark Microplate Absorbance Reader (Bio-Rad Laboratories), according to the manufacturer׳s instructions.

### 2.4 Protein sample processing and peptide labelling with isobaric tags for relative and absolute quantification (iTRAQ) peptide labelling reagents

Proteins were precipitated by adding 6 volumes of ice-cold acetone to 150 μg of the protein extract, re-suspended in 20 µl of TEAB (triethylammonium bicarbonate, 1 M, pH 8.5) and denaturated by adding 1 µl of 2% SDS. Cysteines were reduced with 2 µl of tris(2-carboxyethyl)phosphine (TCEP, 50 mM) and alkylated with 1 µl MMTS (s-methyl methanethiosulfonate, 200 mM). Subsequently, the proteins were digested with trypsin as previously described (Pereira 2011). The quality and amount of proteins and the efficiency of the trypsin digestion were controlled by analysing 20 µg of protein extract in a 10% acrylamide gels. The iTRAQ labelling of the digests, and the combining of the labelled digests into one sample mixture was performed using the manufacturer׳s protocols (iTRAQ^®^ Reagents – 8-plex, AB SCIEX^™^, Framingham, MA, USA). iTRAQ labelling efficiency was 95.1% for iTRAQ study 1 (Cu^2+^) and 95.9% for iTRAQ study 2 (Cd^2+^). Combined samples were concentrated by vacuum (Eppendorf, Hamburg, Germany).

### 2.5 High-resolution hydrophilic interaction chromatography (HILIC) fractionation

Samples were resuspended in HILIC buffer A (10 mM NH_4_HCO_2_, 80% ACN, pH 3.0) and fractionated by HILIC using a PolyHydroxyethyl^™^ A column (PolyLC, Columbia, MD, USA) with 5 μm particle size, 20 cm length×2.1 mm diameter and 200 Å pore size on a Ultimate 3000 HPLC (Thermo Scientific, formerly Dionex, Amsterdam, The Netherlands) controlled by Chromeleon Software, version 6.5 (Thermo Scientific). A set of binary gradient buffers was used for liquid chromatography: buffer A (see above) and buffer B (10 mM NH_4_HCO_2_, 5% ACN, pH 4.0). The binary gradient began with 0% B for 10 min, followed by a linear ramp from 0 to 60% B for 30 min, an extended ramp from 60 to 100% B for 5 min, a further isocratic wash 100% B for 10 min, and column re-equilibration at 0% B for 1 min, in a total of 66 min. Injection volume was set at 20 μl with a constant chromatographic flow rate of 0.5 ml/min. Fractions were collected using a Foxy Jr. Fraction Collector (Dionex, Sunnyvale, CA, USA) in 30 s intervals across 60 min, while the chromatogram was monitored at a wavelength of 280 nm. The fractions were cleaned using C18 UltraMicroSpin Columns (The Nest Group Inc., Southborough, MA, USA) according to the manufacturer׳s guidelines, prior to vacuum centrifugation (Eppendorf).

### 2.6 Reverse phase liquid chromatography (RPLC)–MS analysis

RPLC analysis was performed using an Acclaim^R^ PepMap100 C18 column (Thermo Scientific) with 3 μm particle size of 15 cm length×75 µm diameter and 100 Å pore size on a Ultimate 3000 HPLC (Dionex), and the MS analysis was performed using QStar XL Hybrid ESI Quadrupole Time-of Flight Mass Spectrometer, ESI-qQ-TOF-MS/MS (AB SCIEX^™^; MDS-SCIEX, Concord, Ontario, Canada). Samples were resuspended in RPLC buffer C (3% ACN and 0.1% TFA), injected and captured onto a 0.3×5 mm pre-analytical trap cartridge (5 μm C18 columns) (Thermo Scientific). Peptides were subsequently eluted using an automated gradient with a flow rate of 03 µl/min. Online nLC was achieved using a 150 min binary gradient with RPLC buffer A (0.1% formic acid and 3% ACN), and RPLC buffer B (0.1% formic acid and 97% ACN). A programmed gradient started with a 20 min linear ramp from 0% to 3% buffer B, 95 min ramp from 3% to 35% buffer B, a 30 s rapid ramp up to 90% buffer B, 6.5 min isocratic wash 90% buffer B, 30 s rapid ramp down to 3% buffer B, followed by 27.5 min isocratic wash 3% buffer B. Data acquisition in the mass spectrometer was set to acquire in the positive ion mode, with the precursor ion scan performed within a range of 330–2000 *m*/*z* and a selected mass detector range of 400–1250 *m*/*z*, on a predefined accumulation time of 1 s (Analyst QS Software, AB SCIEX^™^). During the TOF-MS scan, two dynamically selected precursors with a +2 or +3 charge state were isolated for CID fragmentation. Samples were reanalyzed on a second LC–MS injection with identical parameters to increase sample coverage [3].

### 2.7 MS data analysis

Peak list conversion was performed using the mascot.dll embedded script (V1.6) coupled with Analyst QS 1.1.1 (AB SCIEX^™^) with MS/MS group summations and the iTRAQ region deisotoping removed. Protein identification and quantification was carried out in Phenyx v2.6 (GeneBio S.A., Geneva, Switzerland), using a database comprising all *Cyanothece* sp. CCY 0110 protein sequences obtained from UniProt (6413 entries retrieved, March 2014). General search parameters allowed for MS and MS/MS tolerance up to 0.1 Da and one missed cleavage. Fixed protein modifications included iTRAQ lysine and iTRAQ N-terminus (+304 Da) and methyl-thiol of cysteins (+46 Da), and the oxidation of methionine (+16 Da) was defined as variable modification. Acceptance threshold for peptide identification was set at peptide length ≥6, *z*-score ≥5 and *p*-value ≤1 e-4. False discovery rate (FDR) was calculated using a decoy database automatically created by reversing the sequences from the target database, and only proteins satisfying a 1% FDR and identified with at least two peptides unique were considered for further quantitative analysis. iTRAQ labelling efficiency was calculated using peptide data where iTRAQ lysine and iTRAQ N-terminus (+304 Da) modifications were set as variable instead of fixed, and was 95.1% and 95.9% for the copper and cadmium data sets respectively. Since iTRAQ ratios and determination of proteins altered between samples, it was carried out an in house data analysis pipeline [4] by which protein quantifications were obtained by computing the geometric means of the reporters׳ intensities. Median correction was subsequently applied to every reporter in order to compensate for systematic errors. These factors, estimated at the protein level, are used in subsequent analysis. The reporters׳ intensities, in each individual MS/MS scan, were then themselves median corrected using the same factors. Since two replicates are available for each condition, a change is reported only if it is significant regardless of which replicate is chosen to perform the t test comparison. Proteins were subsequently organised into functional groups according to their Gene Ontology information available in Uniprot (http://www.uniprot.org/).

### 2.8 Statistical analysis

To investigate the groups of proteins with similar variation of its relative levels in the different phenotypes, a hierarchical cluster analysis was performed. For that, protein ratios were transformed into ordinal/ranked variables according to their values, namely: 0 – significant fold change <1, 1 – no significant fold change, 2 – significant fold change >1 and clustered using the “Centroid Linkage” method and the “Squared Euclidean Distance” measure. The cluster analysis was performed using the IBM^®^ SPSS^®^ Statistics 20.0 (IBM, Armonk, NY, USA).

## References

[1] Mota R., Pereira S.B., Meazzini M., Fernandes R., Santos A., Evans C.A., De Philippis R., Wright P.C., Tamagnini P. (2015). J. Proteomics.

[2] Rippka R., Deruelles J., Waterbury J.B., Herdman M., Stanier R.Y. (1979). J. Gen. Microbiol..

[3] Chong P.K., Gan C.S., Pham T.K., Wright P.C. (2006). J. Proteome Res..

[4] Pham T.K., Roy S., Noirel J., Douglas I., Wright P.C., Stafford G.P. (2010). Proteomics.

